# Feasibility of e-Health Interventions on Smoking Cessation among Vietnamese Active Internet Users

**DOI:** 10.3390/ijerph15010165

**Published:** 2018-01-20

**Authors:** Bach Xuan Tran, Xuan Thanh Thi Le, Phuong Ngoc Nguyen, Quynh Ngoc Hoang Le, Hue Thi Mai, Huong Lan Thi Nguyen, Huong Thi Le, Tung Thanh Tran, Carl A. Latkin, Melvyn W.B. Zhang, Roger C.M. Ho

**Affiliations:** 1 Institute for Preventive Medicine and Public Health, Hanoi Medical University, Hanoi 100000, Vietnam; nguyenngocphuong2905@gmail.com (P.N.N.); huemt93@gmail.com (H.T.M.); lethihuong@hmu.edu.vn (H.T.L.); 2 Bloomberg School of Public Health, Johns Hopkins University, Baltimore, MD 21205, USA; carl.latkin@jhu.edu; 3 Vietnam Young Physicians’ Association, Hanoi 100000, Vietnam; 4 Faculty of Pharmacy, Duy Tan University, Da Nang 550000, Vietnam; quynhle2k01@gmail.com; 5 Institute for Global Health Innovations, Duy Tan University, Da Nang 550000, Vietnam; huong.ighi@gmail.com (H.L.T.N.), tung.ighi@gmail.com (T.T.T.); 6 Biomedical Global Institute of Healthcare Research & Technology (BIGHEART), National University of Singapore 117599, Singapore; ciezwm@nus.edu.sg; 7 Department of Psychological Medicine, Yong Loo Lin School of Medicine, National University of Singapore, Singapore 119228, Singapore; hocmroger@yahoo.com.sg

**Keywords:** feasibility, ehealth, intervention, smoking, Vietnam, youth

## Abstract

*Introduction:* Although e-health interventions are widely implemented as a supportive measure to smoking cessation, there is a lack of evidence in the feasibility of its application among Vietnamese youths, which is considered to be one of the most frequent internet using populations. This study assessed the quitting attempts among smokers and their preference and willingness to pay for smartphone-based cessation supporting applications in a sample of active internet users approached. *Methods:* A total of 1082 participants were recruited for the online-based survey from August to October 2015 in Vietnam. Information on sociodemographic characteristics, health information seeking behaviors on the internet, smoking status, quitting attempts and willingness to pay for smartphone-based cessation supporting applications were collected. Multivariate logistic regression was used to determine the associated factors with current smoking and willingness to pay for the smoking cessation application. *Results:* About 11% of participants were current smokers while 73.4% had attempted to quit smoking. Only 26.8% of the individuals indicated that they were willing to utilize a smartphone application to assist them in quitting. Participants who were male, had partners/spouse and lived at other places were more likely to smoke cigarette. Meanwhile, people who spent 50–70% of their online time to read health information were less likely to smoke. Results also show that living with family and never sharing health information on the internet were negatively associated with a participant’s willingness to pay for the smartphone application. Meanwhile, people who highly trusted health information were more likely to be willing to pay for the application. *Conclusions:* This prevalence of smoking and associated factors can provide potential indicators for creating several public health interventions in the new environment with the increasing development of information technology. This study implies that in order to expand the coverage of smoking cessation interventions, we recommend the integration of e-health interventions with clinical- or telephone-based conventional models by providing smartphone applications and information on the internet from reliable sources.

## 1. Introduction

Smoking results in significant morbidity, with approximately 6 million deaths occurring annually due to complications [1]. In Vietnam, tobacco smoking is still a public health crisis. Recent reports indicated that the overall percentage of smokers in Vietnam was at 22.5% (45.3% among male and 1.1% among females) [2]. Although the recent prevalence of tobacco smoking is lower than it was in 2010 (47.4% men and 1.4% women), 2006 (49.2% and 1.5%, correspondingly) and 2001 (with 56.1% and 1.8%, respectively) [2,3], there hasn’t been a significant change in the total number of smokers: from 15.5 million smokers in 1997 to 15.3 million in 2010 [2,4]. Many regulations and campaigns have been issued by the Vietnamese government to combat this epidemic such as educational campaigns, graphic health warnings, higher taxes on tobacco products, and the establishment of smoking cessation programs [3], yet the reduction in the smoking rate has been insignificant. Among youths people aged 15–24, it is estimated that more than 2 million people smoked some type of tobacco product [2]. A larger body of literature supports the notion that earlier age of initial smoking was linked to more cigarette use and worse tobacco-related health outcomes in adulthood [4,5], and lower likelihood of quitting smoking [5]. Therefore, it is essential to propose a new approach to alleviate harms of tobacco use in the youth group. 

With the increasing accessibility to smartphones and the internet, people today have greater availability to information. However, the benefits of these technologies in terms of smoking behaviors are still debated. A prior study has emphasized that among adolescents, online social networking sites might facilitate them to engage in smoking behaviors [6]. Another study showed that adolescents who were exposed to images of their peers smoking were more likely to pick up the habit of tobacco smoking [7]. Otherwise, social media and other digital platforms could complement the traditional method in helping young people quitting smoking [8]. A trial of a digital multi-media smoking cessation intervention suggested that those who received the internet- and cell-phone-based intervention experienced more quitting attempts than the control group who only received a self-help booklet [9]. Another review suggested that those received automated email feedback were more likely to improve smoking behaviors [10]. 

Though there have been previous studies about the use of e-health interventions, studies specifically focusing on Vietnamese youths, have not been well documented. This study assessed the quitting attempts among smokers and their preference and willingness to pay for smartphone-based cessation supporting apps through a sample of active internet users approached via Facebook. The findings are pertinent as it will provide a scientific basis for government policies for expanding the coverage and increasing the effectiveness of quitting cessation programs.

## 2. Materials and Methods

### 2.1. Study Design and Sampling Technique

This anonymous online cross-section survey was conducted from August to October 2015 in Vietnam via Google Form (https://www.google.com/forms/about/). Recruitment advertisements were posted in social networking sites (e.g., http://www.facebook.com) and online forums of several high schools and universities including Hanoi Medical University, Vietnam National University, Phan Boi Chau high school, and Hung Yen high school. These selected groups show the diversity of age, gender, living location, and levels of education represented in the study. The advertisement included a brief introduction of the survey and a link to it. We selected people who met inclusion criteria: (1) Aged from 15 to 25; (2) Currently living in Vietnam; (3) Agree to participate in this study. A total of 1082 youths agreed to participate in this study.

### 2.2. Measurements

Web-based questionnaires were implemented using Google Form. Prior to the participants’ participation in the study, they were given the study’s purpose, methods and information of the investigators. The first page also contained several questions used to screen eligible participants. The entire survey took 15–20 min to complete. 

The web-based survey was piloted with twenty youths of different ages and genders. They assessed the usage of this platform and provided recommendations with regards to the further optimization of the online survey. The logical check was applied to ensure that the information that the participants entered was valid. 

We asked participants to report whether they were a current smoker or not. They were classified “current smokers” if they answered “Yes” for the question, “Have you ever smoked tobacco in the last 30 days?” We collected data about age of initial smoking, days of smoking in the last 30 days and number of cigarette smoking per typical days. In terms of smoking cessation, we asked respondents to report their stages of quitting smoking (based on the Transtheoretical model [11,12]), comprising: (1) Pre-contemplation, (2) Contemplation, (3) Preparation, (4) Action, and (5) Maintenance. Participants were asked a following question if they were current smokers: “Are you thinking about quitting smoking?” with four options: “No thought of quitting”, “Think I should quit, but not quite ready”, “Starting to think about how to change my smoking behavior”, “Currently taking action to quit”, and “Already maintaining cessation from smoking”, which reflect the stages (1) to (5), respectively. 

Preferred methods to quit and willingness to pay for smartphone application to motivate smoking quitting were also collected. We asked the respondents to answer two questions: “Which methods do you prefer to use if you quit smoking?” and “Are you willing to pay for a smartphone application that will help you quit smoking?”

Information on internet use was collected by using the following questions: “How many hours do you spend on the internet per day?”; “How much time do you spend reading health information on the Internet?”; “Are you interested in health-related information shared on the internet?”; “How reliable is the health information shared on the internet?”; “Have you shared the health information posted on the internet?”; “Do you follow the health-related information shared on the internet?”. 

### 2.3. Statistical Analysis

Data analysis was performed by using STATA 12.0 software (Stata Corp. LP, College Station, TX, USA). Multivariate logistic regressions were employed to identify the associated factors with two dependent variables: current smoker (Yes/No), and willingness to pay for the smartphone application to motivate the smoking quitting (Yes/No). Independent variables included: sociodemographic characteristics, the interest in/the trust in/sharing health information on the internet, time spent on reading health information when using the internet, and the usefulness of health information on the internet. We applied a stepwise forward model strategy which used the log-likelihood ratio test at a *p*-value of 0.2 to select variables for the reduced models. A *p*-value of less than 0.05 was set as the level of statistical significance.

## 3. Results

Table 1 lists the socioeconomic characteristics of the participants. About 41.9% of participants were male, 65% were over 22 years old, 72.6% were single and 47.5% lived at a homestay.

Table 2 shows that the minority of respondents interested in and highly trusted the health information on the Internet (19.5% and 17.5% correspondingly). Most of them found that the health information on the Internet were not either useful or useless. Furthermore, 32.4% never shared health information on the Internet, and 12.3% never practiced the health information on the Internet.

Table 3 presents that 11.0% of the participants were currently smoking. Among those who smoked, approximately 46.6% of smokers began smoking under the age of 18. The vast majority (59.5%) of smokers have spent at least 10 days smoking on average in the past month, and most of these smokers have been smoking less than 10 cigarettes per day (69.8%). Regarding quitting attempts, 27.6% of current smokers did not want to quit smoking (pre-contemplation stage), while 23.4% had taken action to quit, and 30.6% were maintaining their abstention from smoking. Regarding their preferred method of quitting smoking, the use of chewing gum was the most preferred choice (14.7%), followed by having support from relatives (9.5%) and friends (8.6%). Only 26.8% of the individuals indicated that they were willing to utilize a smartphone application to assist them in quitting.

Table 4 displays the factors associated with smoking among respondents. Participants who were male, had partners/spouse and were living at other places were more likely to smoke cigarettes. Meanwhile, participants who spent 50–70% of their online time to read health information were less likely to smoke.

Table 5 illustrates factors associated with the willingness to pay for smoking-cessation application. The result also shows that living with family and never sharing health information on the internet were both negatively associated with the willingness to pay for the smartphone application to assist with quitting smoking. Meanwhile, people who highly trusted health information were more likely to be willing to pay for the application.

## 4. Discussion

This study partly contributed to the understanding of the feasibility of e-health interventions on smoking cessation among youths in developing countries such as Vietnam. We found that the use of e-health interventions had potential among youths for smoking cessation since many of them are active internet users and perceived internet health information to be useful. About one fourth of smokers were willing to pay for a smoking-cessation application, and participants who believed that health information on the internet was reliable, were more likely to be willing to pay for the application.

The result has indicated that 11% of internet users smoked, which was slightly lower than in the smoking rate reported among people aged 14–24 (12.6%) in Vietnam [2]. Notably, at least 26.8% of the sampled individuals who smoked perceived that a smartphone application was suitable to help support smoking cessation and willing to pay for this application. With the rapid advancement and developments of mobile health over the past decade, there have been a variety of smoking cessation applications that are both theory driven and integrated using behavioral change models. The effectiveness of these applications has been proven in many studies [13,14,15]. Therefore, it is necessary to develop Vietnamese friendly applications for supporting tobacco cessation and encourage certified health providers to apply these applications.

After adjusting potential confounders, we found that people who highly trusted health information on the internet were more likely to be willing to pay for the smartphone application. Moreover, individuals who never shared health information on the internet were less likely to be willing to pay. These results imply the importance of reliable sources for health information on the internet. If youths can access reliable information, it may facilitate their smartphone usage and increase the feasibility of mobile health interventions on smoking cessation [16]. 

Several implications could be suggested based on the results. First, it is essential to increase the accessibility of reliable health information on the internet. From the perspective of the general population, individuals need to protect themselves from misinformation by finding mainstream and validated websites. From news providers’ perspective, health information should be based on scientific findings, and approved by health experts. Thus, it may result in an increase in the trust of the general population, and thereby enhancing the feasibility of eHealth and mobile health interventions on smoking cessation. Second, while it is feasible to apply e-health approach for smoking cessation campaigns, several preparation steps should be done to ensure the success of this new platform including the availability, the cost, and the effectiveness of smoking cessation applications on the smartphone. 

There are several limitations in our study. Given its internet-based sampling approach, findings of this study might not be generalizable to the general population, as the sociodemographic of these individuals differed from the general population. A further study among smartphone users should be conducted to measure how they perceived the benefits of smartphone applications for smoking cessation, which may give us more in-depth results than just examining the general internet population. In particular, our participants were required to have access to an internet connection in order to fill out the respective survey. Moreover, we collected data based on self-reported questions, which may result in bias. 

## 5. Conclusions

In conclusion, this prevalence of smoking and associated factors will provide potential indicators for creating several public health interventions in the new environment with the sharp development of information technology. This study implies that in order to expand the coverage of smoking cessation interventions, we recommend the integration of e-health interventions with clinical- or telephone-based conventional models by providing smartphone applications and information on the internet from reliable sources.

## Figures and Tables

**Table 1:** Demographic characteristics of internet respondents (*n* = 1082).

Characteristics	*n*	%
**Gender**		
Female	628	58.2
Male	452	41.9
**Age groups**		
<18	18	1.7
18–22	358	33.3
>22	699	65.0
**Education attainment**		
<High school	86	8.0
High school	63	5.8
Vocational training/College	830	76.7
Undergraduate	103	9.5
**Marital status**		
Single	786	72.6
Have partners	248	22.9
Have spouse	48	4.4
**Current living location**		
Homestay	514	47.5
Dormitory	130	12.0
Living with family	335	31.0
Living with relatives	87	8.0
Others	16	1.5

**Table 2:** Heath information seeking behavior among internet respondents (*n* = 1082).

Characteristics	*n*	%
**Interested in health information on internet**		
Interest	205	19.5
Neutral	555	52.7
Less interest	257	24.4
No interest	36	3.4
**Time spent reading health information when using internet**		
<10%	312	29.7
10–30%	368	35.1
30–50%	182	17.3
50–70%	87	8.3
70–100%	10	1.0
Unknown	91	8.7
**Trusted in health information on internet**		
Unreliable (<10%)	88	8.1
Low reliable (10–30%)	208	19.2
Normal (31–50%)	547	50.6
Highly reliable (51–70%)	161	14.9
Very highly reliable (>70%)	28	2.6
Unknown	50	4.6
**Share health information on internet**		
Never	337	32.4
Sometimes	621	59.7
Often	65	6.2
Always	18	1.7
**Follow health advice on internet**		
Never	134	12.8
Sometimes	828	78.9
Often	80	7.6
Always	8	0.8
**Usefulness of health information on internet**		
Very useless	10	1.0
Useless	43	4.1
Normal	629	60.0
Useful	318	30.3
Very useful	48	4.6
	**Mean**	**SD**
**Daily time using internet (Hour/day)**	3.5	7.2

**Table 3:** Smoking and quitting behaviors among smoking respondents (*n* = 1082).

Characteristics	*n*	%
**Current smoker**		
Yes	116	11.0
No	942	89.0
**Age of initial smoking**		
<18	45	46.6
18–22	50	43.1
>22	12	10.3
**Days of smoking in the last 30 days**		
≤10	69	59.5
11–20	9	7.8
21–30	38	32.8
**Number of cigarette smoking per typical days**		
≤10	81	69.8
11–20	20	17.2
>20	15	12.9
**Stages of quitting smoking**		
Pre-contemplation	27	27.6
Contemplation	13	13.3
Preparation	5	5.1
Action	23	23.4
Maintain	30	30.6
**Preferred method to quit**		
Use chewing gum	17	14.7
Support from health staff	0	0.0
Support from relatives	11	9.5
Support from friends	10	8.6
Nicotine replacement therapy	0	0.0
Notification from mobile phone	3	2.6
**Willingness to pay for smoking-cessation application**	22	26.8

**Table 4:** Associated factors with current smoking (*n* = 1082).

Associated Factors	Current Smoking		
	OR	95% CI	
**Age (Male vs. Female)**	13.52 *	7.14	25.62
**Age group (vs. <18 years)**			
>22 years	1.52	0.85	2.69
**Marital status (vs. Single)**			
Having partners	2.24 *	1.33	3.78
Having spouse	5.91 *	2.37	14.71
**Living location (vs. Homestay)**			
Living with family			
Living with relatives	0.24	0.06	1.05
Others	5.13 *	1.10	23.98
**Time spent on reading health information when using internet (vs. <10%)**			
50–70%	0.22 *	0.05	1.00
**Share health information on internet (vs. Always)**			
Sometimes	0.65	0.41	1.05
Never			
**Usefulness of health information on internet (vs. Very useful)**			
Useful	1.60	0.97	2.65
* *p* < 0.05			

**Table 5:** Associated factors with the willingness to pay for smoking-cessation application (*n* = 116).

Associated Factors	Willing to Pay for Smoking-Cessation Application		
	OR	95% CI	
**Education (<High school)**			
Undergraduate	0.21	0.03	1.28
**Living location (vs. Homestay)**			
Living with family	0.12 *	0.02	0.73
Living with relatives			
Others			
**Interested in health information on the internet (vs. Interest)**			
Less interest	3.30	0.82	13.33
**Trusted in health information on internet (vs. Normal)**			
High reliable	7.20 *	1.20	43.09
Unknown	43.35 *	2.29	821.2
**Share health information on internet (vs. Always)**			
Sometimes			
Never	0.15 *	0.03	0.91
* *p* < 0.05			
